# Identification of hub genes related to metastasis and prognosis of osteosarcoma and establishment of a prognostic model with bioinformatic methods

**DOI:** 10.1097/MD.0000000000038470

**Published:** 2024-06-07

**Authors:** Zheng Fu, Guofeng Sun, Jingtian Li, Hongjian Yu

**Affiliations:** a Department of Orthopedics, Binzhou People’s Hospital, Binzhou,China; b Department of Orthopedics, the First Affiliated Hospital of Chongqing Medical University, Chongqing, China; c Orthopedic Laboratory of Chongqing Medical University, Chongqing, China.

**Keywords:** bioinformatics, differential analysis, osteosarcoma, prognostic model, survival analysis

## Abstract

Osteosarcoma (OS) is the most common primary malignant bone tumor occurring in children and adolescents. Improvements in our understanding of the OS pathogenesis and metastatic mechanism on the molecular level might lead to notable advances in the treatment and prognosis of OS. Biomarkers related to OS metastasis and prognosis were analyzed and identified, and a prognostic model was established through the integration of bioinformatics tools and datasets in multiple databases. 2 OS datasets were downloaded from the Gene Expression Omnibus database for data consolidation, standardization, batch effect correction, and identification of differentially expressed genes (DEGs); following that, gene ontology and Kyoto Encyclopedia of Genes and Genomes (KEGG) pathway enrichment analyses were performed on the DEGs; the STRING database was subsequently used for protein-protein interaction (PPI) network construction and identification of hub genes; hub gene expression was validated, and survival analysis was conducted through the employment of the TARGET database; finally, a prognostic model was established and evaluated subsequent to the screening of survival-related genes. A total of 701 DEGs were identified; by gene ontology and KEGG pathway enrichment analyses, the overlapping DEGs were enriched for 249 biological process terms, 13 cellular component terms, 35 molecular function terms, and 4 KEGG pathways; 13 hub genes were selected from the PPI network; 6 survival-related genes were identified by the survival analysis; the prognostic model suggested that 4 genes were strongly associated with the prognosis of OS. DEGs related to OS metastasis and survival were identified through bioinformatics analysis, and hub genes were further selected to establish an ideal prognostic model for OS patients. On this basis, 4 protective genes including TPM1, TPM2, TPM3, and TPM4 were yielded by the prognostic model.

## 1. Introduction

Osteosarcoma (OS) is a primary malignant bone tumor of mesenchymal origin, most frequently occurring in children and adolescents.^[1,2]^ Predilection sites include the distal femur, proximal tibia, and proximal humerus,^[3]^ and metastases largely emerge in the lungs.^[4,5]^ The metastasis and relapse of OS are noted to be strongly associated with its poor prognosis.^[6,7]^ Therefore, an enhanced understanding of the pathogenesis and metastatic mechanism of OS on the molecular level might facilitate the identification of diagnostic and therapeutic targets to improve the treatment and prognosis of OS.

With the recent advances in bioinformatics tools and high-throughput sequencing technology, the mechanisms of cancer development and metastasis have been extensively studied, including those responsible for OS development and metastasis.^[8,9]^ High-throughput platform assay technology has been widely employed to identify biomarkers suitable for disease screening, early diagnosis, prognosis, personalized prevention and treatment.^[10–14]^ However, researchers are likely to obtain false-positive and false-negative results due to sample heterogeneity, choice of screening techniques, and coupling effect in independent studies with a small sample size. With integrated analysis based on multiple datasets effectively overcoming the above-mentioned limitations, open-source databases such as Gene Expression Omnibus (GEO), TCGA, ICGC have been applied in numerous studies to identify novel biomarkers for cancer diagnosis, treatment, and prognosis.^[15–17]^ Although the molecular biology of OS has been widely studied, most published works are single independent studies. To transcend the limitations of independent studies, this study used bioinformatic tools and multiple datasets obtained from the GEO database and the Therapeutically Applicable Research to Generate Effective Treatments (TARGET) Initiative to analyze and identify biomarkers associated with the metastasis and prognosis of OS and establish a prognostic model.

First of all, two OS metastasis-related datasets were derived from the GEO database for data consolidation, standardization, and batch effect correction; second, differentially expressed genes (DEGs) were identified and underwent gene ontology (GO) and Kyoto Encyclopedia of Genes and Genomes (KEGG) pathway enrichment analyses; subsequently, a predicted protein-protein interaction (PPI) network was constructed using the STRING database (https://string-db.org/) for screening of hub genes; after validation of hub gene expression, a survival analysis was conducted using the TARGET database (https://ocg.cancer.gov/programs/target); following that, the Spearman’s correlation was employed to screen for survival-related genes; finally, a prognostic model was established and evaluated, with the survival-related genes as variables.

## 2. Materials and methods

### 2.1. Data download

Two datasets, i.e., GSE37552 and GSE85537, which had not been analyzed simultaneously in preceding studies, were downloaded from the Gene Expression Omnibus (GEO) database (https://www.ncbi.nlm.nih.gov/geo/).^[18]^ The 2 datasets were examined by the chip-based platform GPL570 (HG-U133_Plus_2) Affymetrix Human Genome U133 Plus 2.0 Array; the GSE37552 dataset consisted of 2 metastatic OS cell lines and corresponding non-metastatic parental cell lines; the GSE85537 dataset contained 3 primary OS tissue samples and 3 OS lung metastasis tissue samples.

### 2.2. Identification of DEGs

GSE37552 and GSE85537 were consolidated before data standardization and batch effect correction. The “limma” R package (version 3.40.2) was applied to analyze differentially expressed mRNAs, with “*P* value ≤ .05 and Log₂ (Fold Change) ≥ 1 (upregulated) or Log₂ (Fold Change) ≤ −1 (downregulated)” being defined as the threshold for differential expression. Boxplots and volcano plots were generated using the ggplot2 package in R; PCA plots were drawn by the ggord package in R; heatmaps were plotted by ComplexHeatmap in R.^[19]^

### 2.3. GO and KEGG pathway enrichment analyses of DEGs

GO analysis was performed on corresponding features of the DEGs identified in terms of using the clusterProfiler R^[20]^ package, while the functional enrichment analysis of DEGs in KEGG pathways was conducted using the same package, where *P*.adj < 0.1 and *q* value < 0.2 were set as the threshold for DEGs of significant enrichment.

### 2.4. Construction of PPI network and identification of hub genes

The STRING database (https://string-db.org/cgi/input.pl) designed for the collection, rating, and integration of all publicly available PPI data sources and data supplementation via computation and estimation to elucidate PPI, including direct (physical) and indirect (functional) associations.^[21]^ The identified DEGs were imported into the STRING database (version 11.5) to establish a PPI network with a confidence cutoff score of 0.7 (high confidence). Cytoscape 3.8.2 (https://cytoscape.org/) is an open-source bioinformatics software platform for the visualization and integration of molecular interaction networks.^[22]^ PPI network data were imported into the software for PPI network plotting. Finally, the Cytoscape plug-in CytoHubba^[23]^ was applied to screen for 30 top hub genes of the PPI network using the Maximal Clique Centrality (MCC) method.

### 2.5. Validation of hub gene expression

For expression profiling of the hub genes in other datasets, the GSE49003 dataset^[24]^ was downloaded from the GEO database (https://www.ncbi.nlm.nih.gov/geo/), and the GPL6947 Illumina HumanHT-12 V3.0 expression beadchip was used for hub gene expression profiling; the dataset contained 2 metastatic OS cell lines and 2 non-metastatic OS cell lines, with each being validated 3 times. The Wilcoxon signed-rank test was conducted to reveal hub gene expression in the GSE49003 dataset, and the results were visualized by generating violin plots using the ggplot2 package in R.

### 2.6. Survival analysis of hub genes

Raw counts of RNA sequencing data and corresponding clinical data of 98 OS cases were downloaded from the TARGET database (https://ocg.cancer.gov/programs/target) for Kaplan–Meier (KM) survival analysis and plotting of KM survival curves using the R package survival and survminer. The KM survival analysis was examined by the Log-rank test for comparison between patients with high and low expression of hub genes; for KM curves, *P* values and hazard ratios (HRs) with a 95% confidence interval (CI) were obtained from the Log-rank test and univariate Cox proportional hazards regression, where *P* < .05 indicated a level of statistical significance.

### 2.7. Spearman’s correlation analysis of hub gene expression

Spearman’s correlation analysis was performed on the raw counts of RNA sequencing data and corresponding clinical data of 98 OS cases from the TARGET database to describe the correlations among non-normally distributed quantitative variables (of hub gene expression), and the significant level was set at *P* < .05. Correlation heatmaps were drawn via the ggplot2 R package.

### 2.8. Construction of prognostic model (gene signature)

Raw counts of RNA sequencing data and corresponding clinical data of 98 OS cases were downloaded from the TARGET database. A prognostic model was constructed using survival-related hub genes as variables. Least Absolute Shrinkage and Selection Operator (LASSO) regression was implemented in combination with 10-fold cross validation for variable screening. The Log-rank test was undertaken to examine the KM survival analysis of survival difference between the high- and low-risk groups, and a time-dependent ROC analysis was performed to compare the model’s predictive accuracy and risk scoring performance for different groups, with *P* < .05 indicative of statistical significance. These analyses were conducted via the glmnet & survival R package and visualized using the ggplot2 R package.

## 3. Results

### 3.1. Identification of DEGs

**Figure 1.**
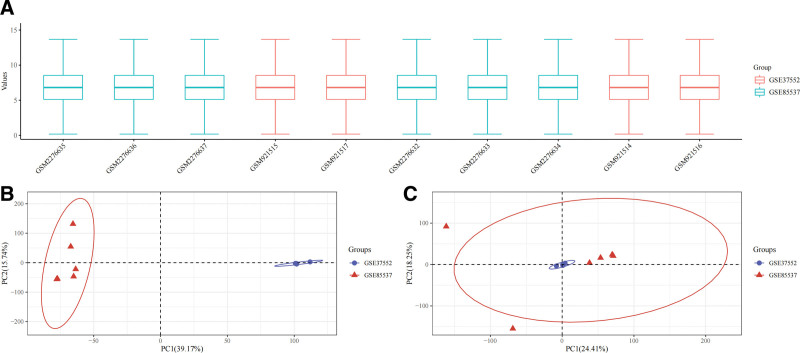
(A) Boxplots after data standardization, with the datasets being denoted in different colors. (B) PCA results before batch effect correction for the 2 datasets separated without any intersection. (C) The PCA results after batch effect correction shows the intersection of the 2 datasets, which can be used as a batch of data for subsequent analysis.

**Figure 2.**
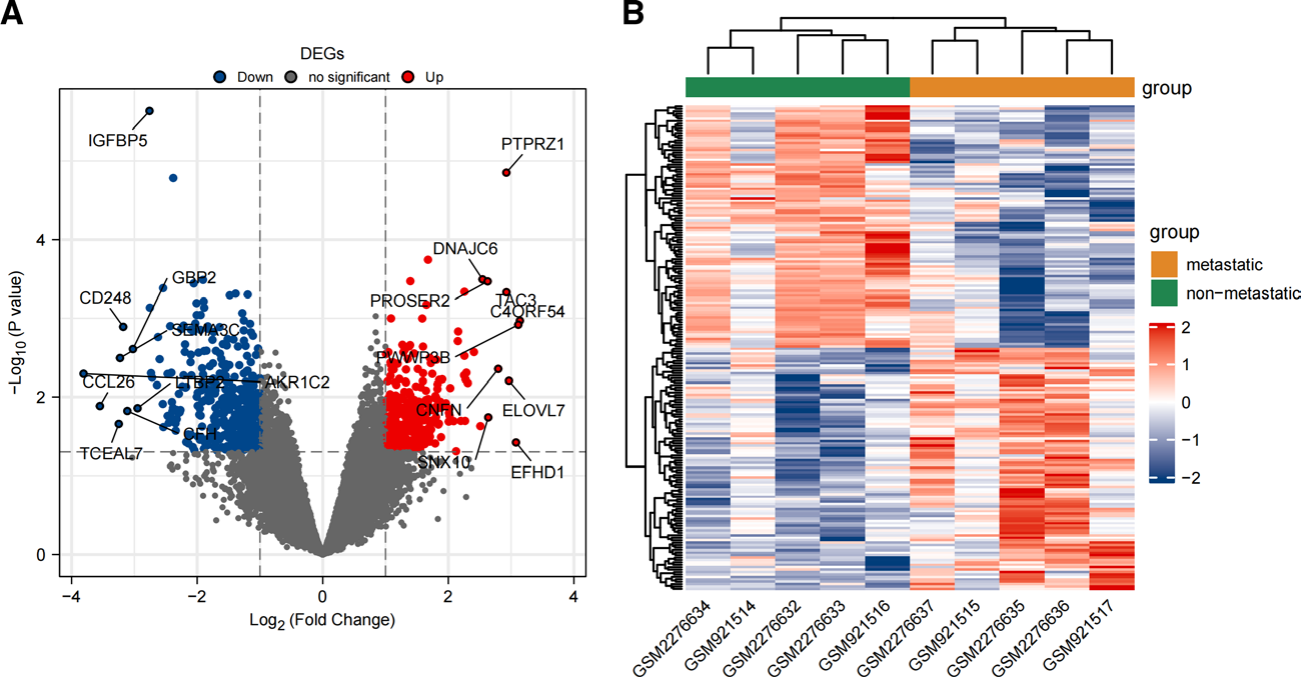
(A) Volcano plots based on Log2 (Fold Change) and −Log10 (*P* value). (B) Heatmap of DEGs based on the hierarchical clustering analysis of DEGs between metastatic and non-metastatic groups, with downregulated genes in blue and upregulated genes in red. DEGs = differentially expressed genes,

The GSE37552 dataset contained expression data of 2 metastatic OS cell lines and corresponding non-metastatic parental cell lines; the GSE85537 dataset consisted of expression data of 3 primary OS tissue samples and 3 OS lung metastasis tissue samples. These datasets were consolidated before standardization and batch effect correction (Fig. 1). A total of 701 DEGs were identified in the differential analysis, including 304 upregulated genes and 397 downregulated genes; results of the differential analysis were depicted as volcano plots, while a heatmap was plotted to display the expression of 100 DEGs with the greatest fold changes (Fig. 2).

### 3.2. GO and KEGG pathway enrichment analysis of DEGs

**Figure 3.**
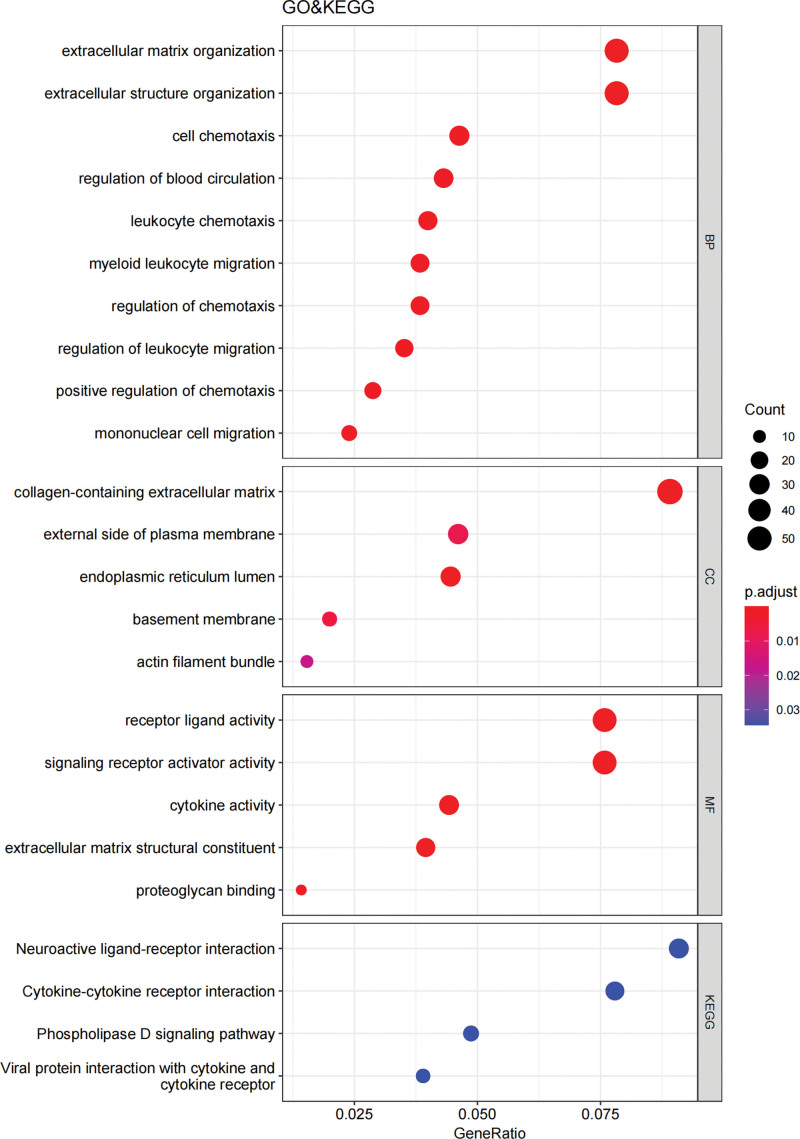
GO and KEGG enrichment analysis of DEGs: The color intensity stands for the adjusted *P* value and the dot size indicates the count of genes assigned to each term. DEGs = differentially expressed genes, GO = Gene Ontology, KEGG = Kyoto Encyclopedia of Genes and Genomes.

**Figure 4.**
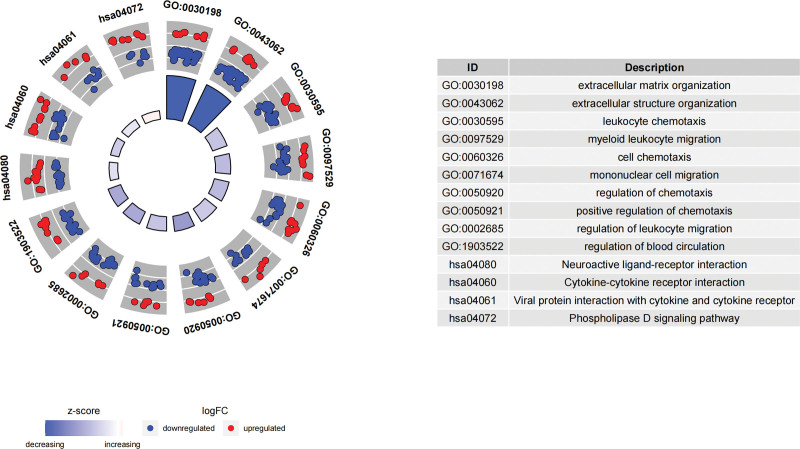
Circle plot: Relationships between the DEGs and different GO or KEGG IDs, with blue dots indicative of downregulated genes and red dots representing upregulated genes. DEGs = differentially expressed genes, GO = Gene Ontology, KEGG = Kyoto Encyclopedia of Genes and Genomes.

By GO and KEGG pathway enrichment analyses, the overlapping DEGs were enriched for 249 BP terms, 13 CC terms, 35 MF terms, and 4 KEGG pathways. Under BP terms, DEGs were basically enriched in the following processes: extracellular matrix organization (GO:0030198), extracellular structure organization (GO:0043062), cell chemotaxis (GO:0060326) and regulation of blood circulation (GO:1903522); as to CC terms, DEGs were mainly enriched in collagen-containing extracellular matrix (GO:0062023), external side of plasma membrane (GO:0009897), endoplasmic reticulum lumen (GO:0005788) and basement membrane (GO:0005604); for MF terms, DEGs were mainly enriched in receptor ligand activity (GO:0048018), signaling receptor activator activity (GO:0030546), cytokine activity (GO:0005125), and extracellular matrix structural constituent (GO:0005201); the KEGG pathway enrichment analysis revealed 4 pathways, namely neuroactive ligand-receptor interaction (hsa04080), cytokine-cytokine receptor interaction (hsa04060), phospholipase D signaling pathway (hsa04072), and viral protein interaction with cytokine and cytokine receptor (hsa04061) (Fig. 3). The circle plot visualizes the relationships between the DEGs and different GO or KEGG IDs (Fig. 4).

### 3.3. Construction of PPI network and identification of hub genes

**Figure 5.**
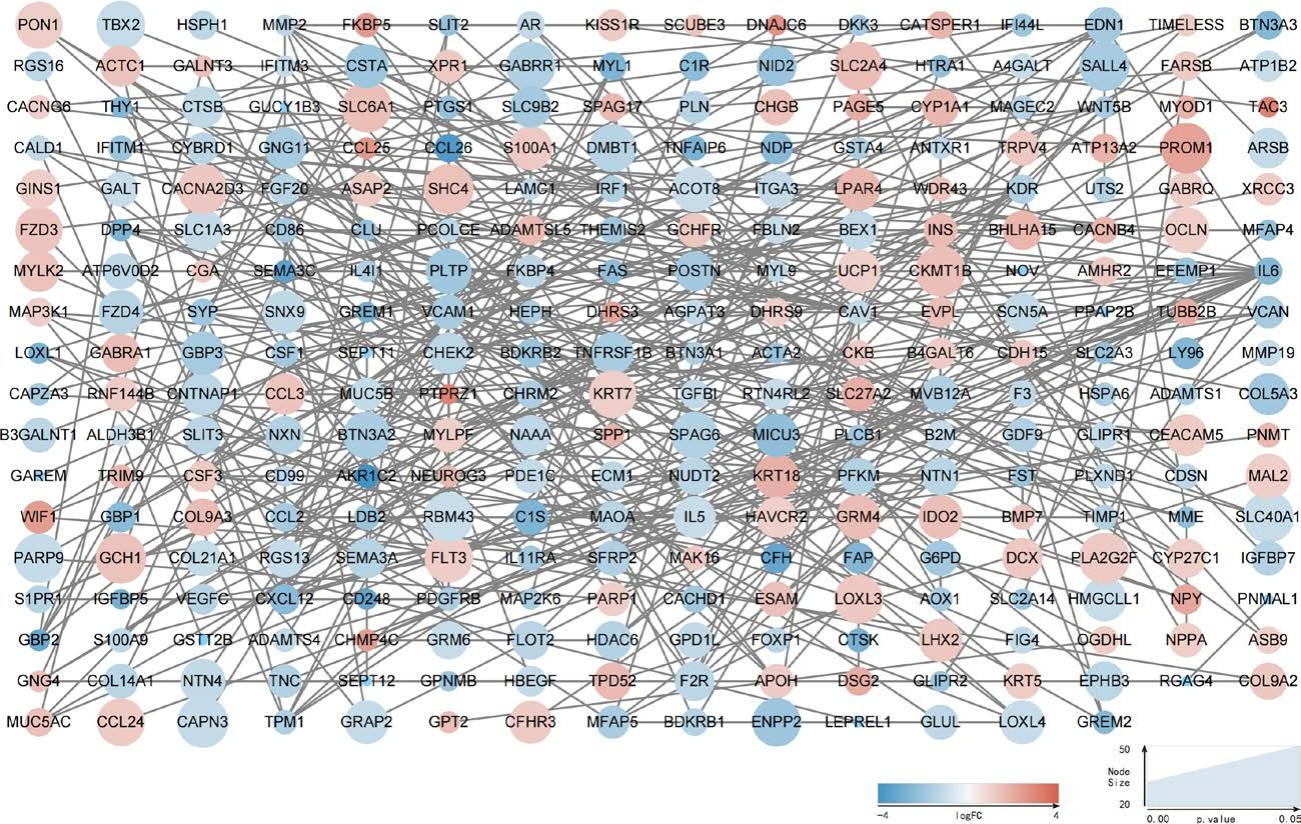
The PPI network: the log2FC of each gene is indicated in gradient color; the *P* value of each gene is indicated by node size. PPI = protein-protein interaction.

**Figure 6.**
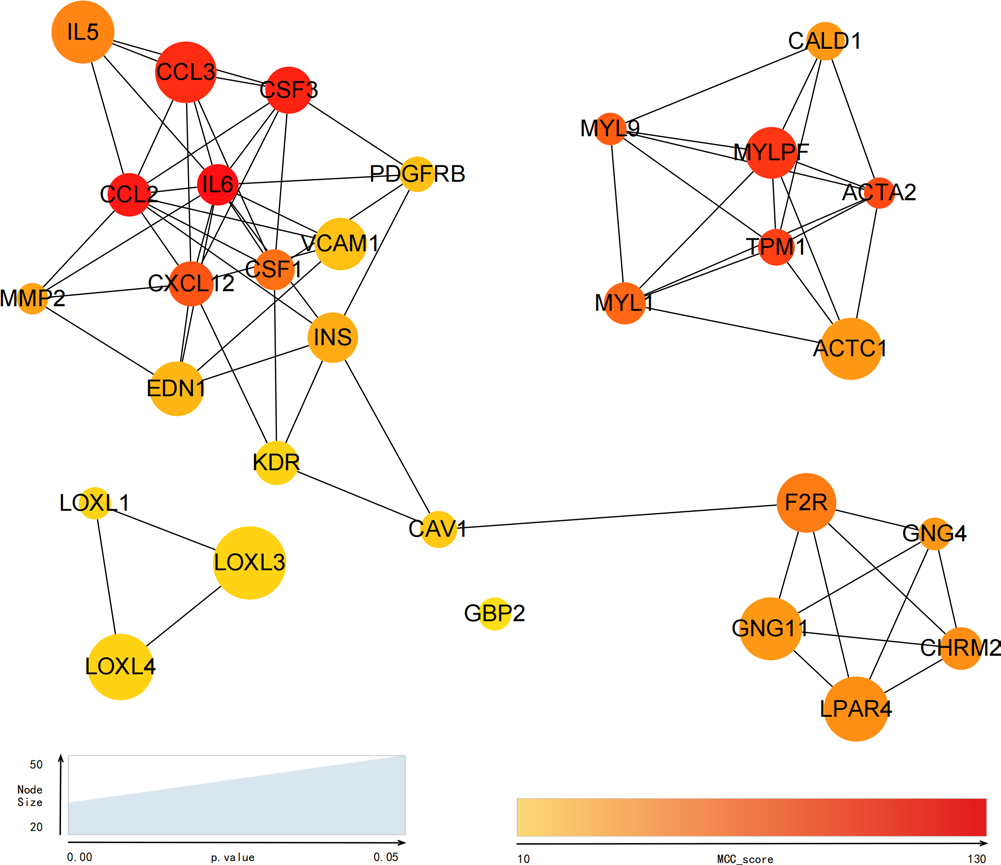
Hub Genes: The top 30 genes ranked by the MCC method are identified as hub genes via the plug-in CytoHubba; the MCC score of each gene is indicated in gradient color; the *P* value of each gene is indicated by node size.

**Figure 7.**
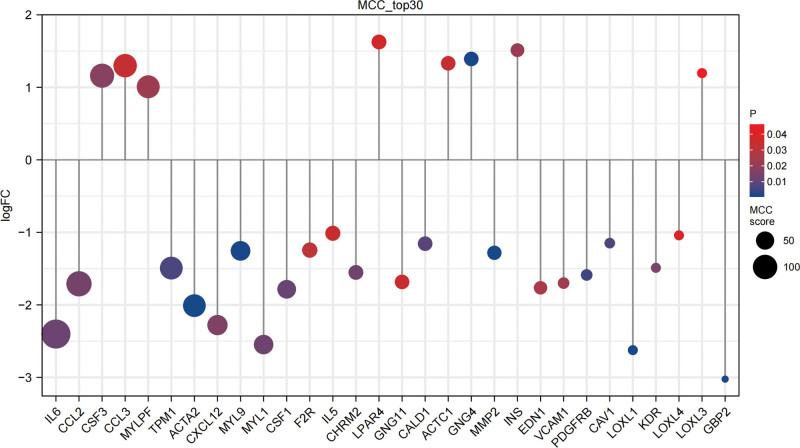
Expression and MCC scores of hub genes: The dot plot has gene name on the x-axis and logFC on the y-axis; the *P* value of each gene is indicated in gradient color; the MCC score of each gene is indicated by dot size. MCC = Maximal Clique Centrality.

A PPI network was constructed to predict DEG-DEG interaction. The PPI network was comprised of 698 nodes and 381 edges, and the average node degree was 1.09 (Fig. 5). Thirty top-ranking genes were identified as hub genes using the MCC algorithm via the plug-in Cytohubba (Fig. 6). The MCC scores and expression of hub genes were displayed by a dot plot (Fig. 7).

### 3.4. Validation of hub gene expression

**Figure 8.**
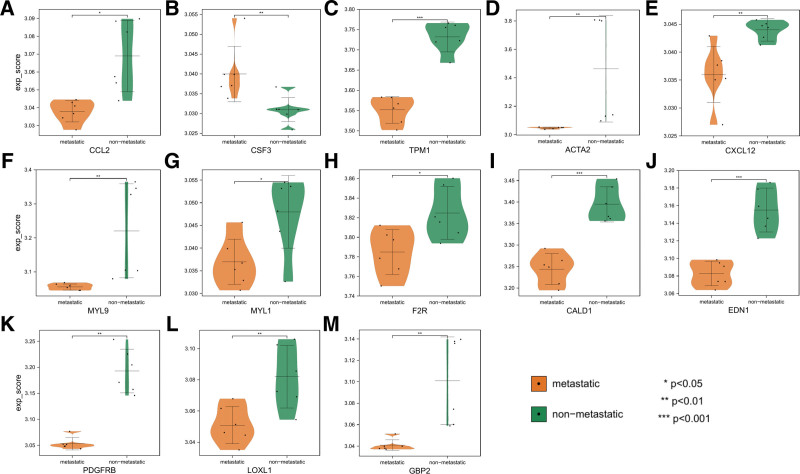
Hub genes in the GSE49003 dataset show significant differences in expression; non-metastatic samples are indicated in green and metastatic samples in orange.

The GSE49003 dataset contained expression profiles of 2 metastatic OS cell lines and 2 non-metastatic OS cell lines that were examined 3 times respectively. To validate the expression of the 30 hub genes, 13 were differentially expressed in GSE49003, including CCL2, CSF3, TPM1, ACTA2, CXCL12, MYL9, MYL1, F2R, CALD1, EDN1, PDGFRB, LOXL1, and GBP2, which conformed to the results yielded by the preceding differential analysis (Fig. 8).

### 3.5. Survival analysis of hub genes

**Figure 9.**
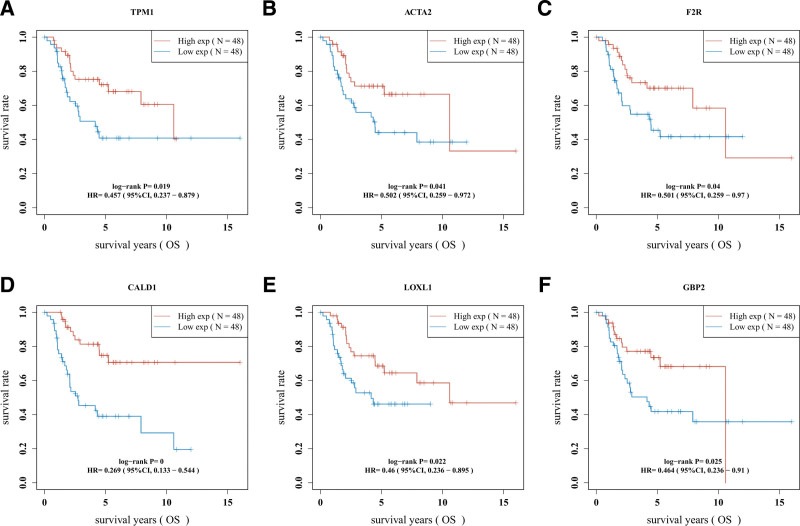
KM survival analysis of the 6 genes: TPM1, ACTA2, F2R, CALD1, LOXL1, and GBP2. KM = Kaplan–Meier.

A survival analysis was performed on the 13 hub genes in the 98 pieces of sample data derived from the TARGET database using KM curves, and 6 DEGs were identified to the survival of OS patients, including TPM1, ACTA2, F2R, CALD1, LOXL1, and GBP2. These 6 DEGs were protective of OS patients, with a high expression level suggesting better survival outcomes (Log-rank *P* < .05) (Fig. 9).

### 3.6. Spearman’s correlation analysis of hub gene expression

**Figure 10.**
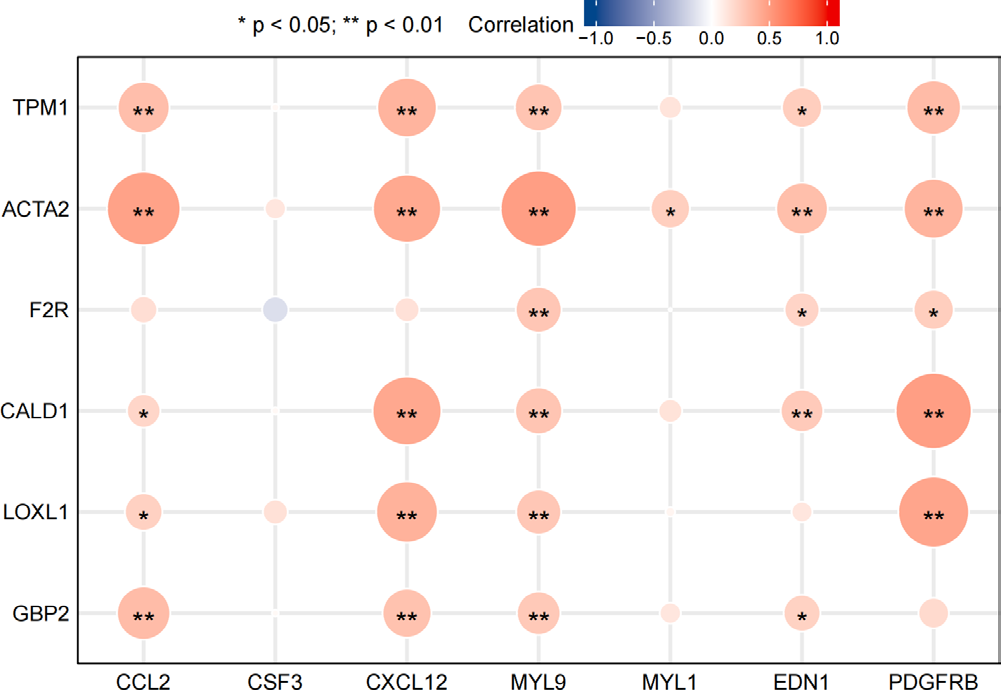
Spearman’s correlation analysis of hub gene expression: The plot has the 6 protective genes on the y-axis and the rest 7 genes on the x-axis; a positive correlation is represented by a red dot and a negative correlation by a blue dot.

The Spearman’s correlation analysis of the 13 hub genes showed that CSF3 had no correlation with the 6 protective genes, while another 6 genes (including CCL2, CXCL12, MYL9, MYL1, EDN1, and PDGFRB) were positively correlated with the 6 protective genes (Fig. 10).

### 3.7. Construction of prognostic model (gene signature)

**Figure 11.**
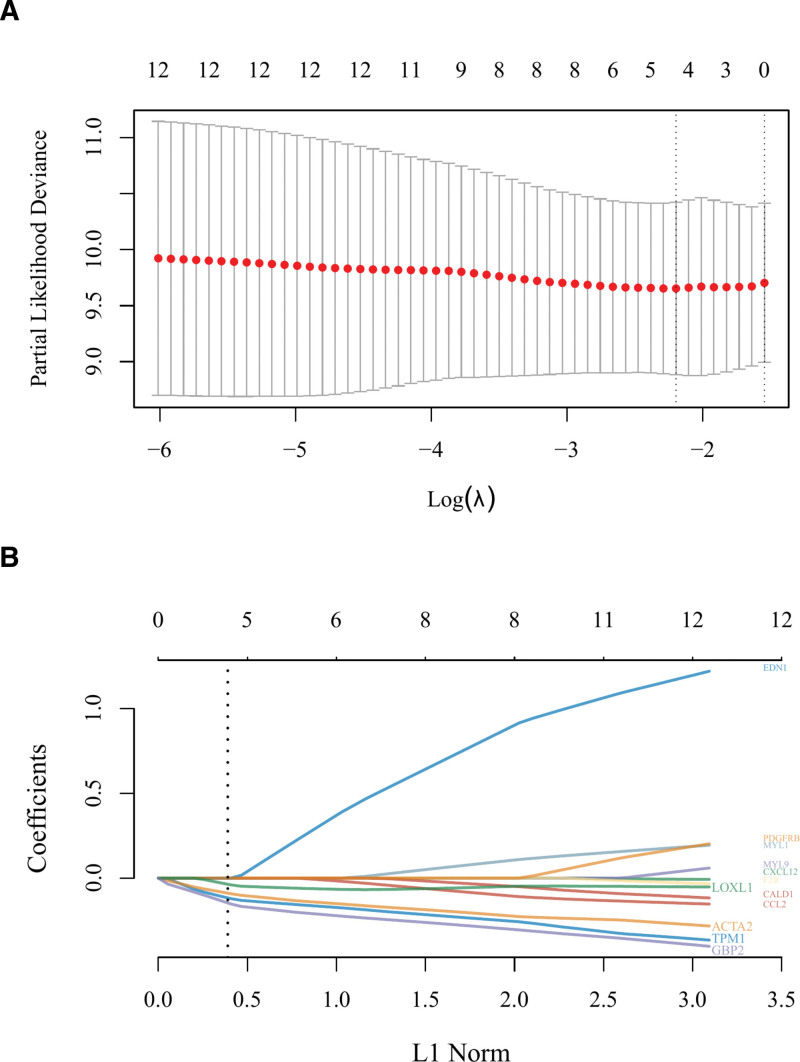
(A) Coefficients of selected features are shown by lambda parameter, partial likelihood deviance versus log(λ) is drawn using a LASSO-Cox regression model. (B) Trajectory of variables (genes) of the prognostic model, with the optimal model having 4 genes, including TPM1, ACTA2, LOXL1, and GBP2, and coefficient value < 0 indicative of protective properties.

**Figure 12.**
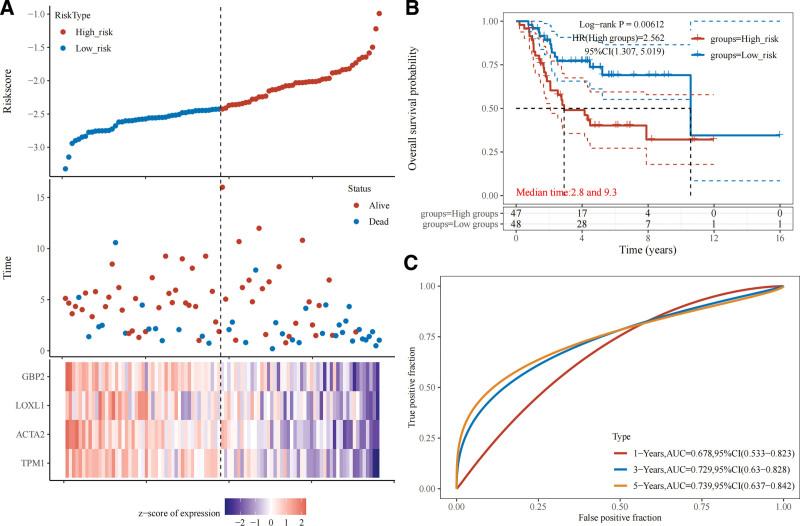
(A) Prognostic analysis of gene signature: the dotted line represents the median risk score and divides the patients into low- and high-risk groups; survival status of the patients, with more dead patients suggestive of a higher risk score; heatmap of the expression profiles of the prognostic genes in the low- and high-risk groups. (B) KM survival analysis of gene signature. (C) Time-dependent ROC analysis of gene signature. KM = Kaplan–Meier, ROC = receiver operating characteristic analysis.

A prognostic model was established using the 12 survival-related hub genes as variables. According to the LASSO-Cox regression model, the optimal model (with the minimal partial likelihood deviance) had a lambda.min of 0.1111 and 4 corresponding variables (genes). The model can be expressed as: Risk score = (−0.1213)*TPM1 + (−0.0925)*ACTA2 + (−0.0402)*LOXL1 + (−0.1527)*GBP2, where the coefficients of the 4 genes, namely TPM1, ACTA2, LOXL1, and GBP2, were all negative in the equation. This revealed the protective properties of the 4 genes for survival, with the absolute value of each coefficient indicative of the weight of each gene (Fig. 11). In the prognostic model, a higher number of OS patients dying in a specific time frame represented a higher risk; the 4 genes were highly expressed in the low-risk group and exhibited low expression levels in the high-risk group; the KM curve indicated a significant difference in survival between the high- and low-risk groups (HR = 2.562, Log-rank *P* = .00612); according to the time-dependent receiver operating characteristic (ROC) analysis, the area under the curve (AUC) corresponding to 1-, 3-, and 5-year survival were 0.678, 0.729, and 0.739, respectively (Fig. 12).

## 4. Discussion

OS is a primary malignant bone tumor most frequently found in children and adolescents, especially in those aged 15 to 19 years, among which the annual incidence rate is 8 to 11 million as the third most commonly occurring tumor in this age group following lymphoma and brain tumor.^[1,2]^ Of mesenchymal origin, OS has a predilection for the distal femur, proximal tibia, and proximal humerus, with over 50% of the patients developing OS around the knee joint; adolescents are most susceptible to OS, which usually starts in the fastest growing areas, suggesting an association with the rapid growth of bones; in few cases, OS occurs as a result of radiant exposure.^[1,3]^ OS exhibits local aggressiveness and early systemic metastasis.^[25]^ Up to 20% to 25% of the OS patients present with metastases at diagnosis, including lung metastases (90%), bone metastases (8–10%), and lymphatic metastases (<2%)^[3–5]^; however, micrometastases are currently difficult to detect in 80% to 90% patients in clinical practice.^[25]^ In addition, OS is recognized as a highly recurrent disease. Reportedly, OS relapses in 30% to 40% of the patients with localized OS; some relapses even occur within 2 to 3 years since the initial treatment, with the lung as the predilection site of metastases.^[25–27]^ Studies show that the metastasis and relapse of OS are closely associated with its poor prognosis; notably, the overall 5-year survival rate for recurrent OS is merely 23% to 29%; in recurrent OS, only 31% of the patients with local recurrence can be cured, and the cure rate is reduced to 10% when the patients present with lung metastases.^[2,6,7,28]^

Multiple efforts have been made to elucidate the molecular biology of OS pathogenesis and metastasis^[8,9,29]^ and form a series of hypotheses. Periodic changes in the PI3K/mTOR pathway and PTEN loss are observed in OS patients^[14,29,30]^; proliferation and early metastasis of OS can be suppressed by inhibiting the Wnt signaling pathway^[31,32]^; overexpression of ΔNp63 occurs frequently in OS, which promotes IL6 production and potentially plays a key role in the mechanism of OS metastasis^[33]^; NF-κB activation affects OS development and progression via the RANK/RANKL pathway.^[34,35]^ However, researchers are likely to obtain false-positive and false-negative results due to sample heterogeneity, choice of screening techniques, and coupling effect in independent studies with a small sample size. Therefore, it is difficult to yield reliable results solely dependent on independent studies. Efforts to study multiple datasets using bioinformatics tools are expected to arrive at an ideal solution for desired results.

To overcome the limitations of the said independent studies, 2 OS datasets derived from the GEO database were studied simultaneously, which involved sample data of metastatic and non-metastatic cell lines, primary tumor, and lung metastases of OS; to achieve desired results, the datasets were analyzed simultaneously after batch effect correction. In the differential analysis, 304 upregulated genes and 397 downregulated genes were identified, and GO and KEGG pathway enrichment analyses were performed on these DEGs, which suggested that under BP terms, DEGs were mainly enriched in an extracellular matrix organization, extracellular structure organization, cell chemotaxis, and regulation of blood circulation, and the enriched KEGG pathways included neuroactive ligand-receptor interaction, cytokine-cytokine receptor interaction, phospholipase D signaling pathway, and viral protein interaction with cytokine and cytokine receptor. Particularly, phospholipase D (PLD) is a member of the phospholipase superfamily, which exists extensively in a wide range of organisms, including mammals.^[36]^ The PLD signaling pathway can be activated by diverse extracellular signals, including growth hormone, insulin, epidermal growth factor (EGF), and vascular endothelial growth factor.^[37]^ Ample evidence reveals the presence of abnormally expressed PLD in many types of cancer, which demonstrates its crucial role in cancer growth and metastasis as well as its potential as a therapeutic target for cancer.^[37–39]^

A PPI network was constructed using the STRING database, and 30 hub genes were identified using the plug-in CytoHubba. The GSE49003 expression profile dataset was downloaded from the GEO database; after validation, the expression levels of the 30 hub genes in GSE49003 were shown to conform to our differential analysis of the 13 genes in GSE40993. The survival analysis using the TARGET database showed that 6 of the 13 genes were strongly associated with the survival of OS patients, including TPM1, ACTA2, F2R, CALD1, LOXL1, and GBP2. The Spearman’s correlation analysis revealed the positive correlations between the genes mentioned above and another 6 genes in the OS cases derived from the TARGET database, namely CCL2, CXCL12, MYL9, MYL1, EDN1, and PDGFRB. These 12 genes were fitted in the LASSO-Cox regression model to obtain the optimal prognostic model: Risk score = (−0.1213)*TPM1 + (−0.0925)*ACTA2 + (−0.0402)*LOXL1 + (−0.1527)*GBP2. Clearly, the survival of OS patients was affected by the 4 genes, with the negative values of the corresponding coefficients indicative of the protective properties of these genes and a high expression level suggestive of a good prognosis; the 4 genes were ranked by weight, which was reflected by the absolute values of corresponding coefficients: GBP2 < TPM1 < ACTA2 < LOXL1. The KM curve of the prognostic model demonstrated its value for prognostic evaluation (HR = 2.562, Log-rank *P* = .00612); The time-dependent ROC analysis (1-year AUC = 0.678, 3-year AUC = 0.729, 5-year AUC = 0.739) showed that the prognostic model could effectively predict the survival rate of OS patients, especially for prediction of the 5-year survival rate.

Guanylate-binding proteins (GBPs) are members of the superfamily of interferoninducible GTPases; as an important player in host immune responses, GBPs display antiviral activity against HIV, hepatitis C virus (HCV), Zika virus, and influenza A virus (IAV).^[40–42]^ Belonging to the GBP family, GBP2 has been widely studied to investigate its role in cancer. A high expression level of GBP2 is found to strongly correlate with a good prognosis of breast cancer and serve as a marker of robust T-cell response^[43]^; GBP2 can suppress mitochondrial fission in breast cancer cells and inhibit breast cancer invasion^[44]^; upregulated GBP2 expression disturbs the Wnt signal to control colorectal cancer cell growth and improve the sensitivity to paclitaxel.^[45]^ Nevertheless, the protective effect of GBP2 is absent in other types of cancer like esophageal squamous cell carcinoma (ESCC), where GBP2 expression is significantly higher than that in normal adjacent tissue^[46]^; GBP2 expression is markedly upregulated in human brain glioblastoma (GBM), which improves the invasion ability of GBM via the Stat3/fibronectin pathway^[47]^; GBP2 expression is significantly higher in pancreatic cancer tissue as compared with normal adjacent tissue and has a close association with a poor prognosis.^[48]^ However, further study is needed as GBP2 expression in OS and the mechanism of how GBP2 affects the development and metastasis of OS have rarely been reported; GBP2 is recognized as a potential diagnostic marker and a therapeutic target for OS. Tropomyosin (TPM) is an actin-binding protein that positions laterally along actin filaments and modulates actin-myosin interaction; as a cytoskeleton stabilizer, TPM is also demonstrated to participate in a series of biological processes, such as cell division, cell movement, cell apoptosis, and signal transduction; besides, abnormal expression of TPM is closely associated with cancer development and progression.^[49,50]^ Four proteins have been identified in the TPM family, including TPM1, 2, 3, and 4. Particularly, TPM1 has been widely recognized as an anti-oncogene that induces cancer cell apoptosis by overexpression.^[51–53]^ In urothelial bladder carcinoma, upregulated TPM1 expression is shown to suppress cancer cell proliferation and cell cycle to accelerate apoptosis, while lnc-RNA MEG3 expression plays a regulatory role in TPM1 expression.^[54]^ In gastric cancer, TPM1 expression is likewise inhibited by miR-183-5p.1, which promotes cancer cell proliferation, migration, and invasion.^[55]^ TPM1 is demonstrated to inhibit lung cancer cell proliferation and invasion and promote cell apoptosis.^[56]^ In line with our analysis of TPM1 in OS patients, these studies reveal the protective effect of TPM1 and highlight the value of further studying the mechanism of action of TPM1 as a potential therapeutic target for OS.

Alpha-smooth muscle actin (α-SMA; hereafter ACTA2) is believed to facilitate the generation of cell mechanical tension, maintain cell shape and movement and potentially play a critical role in cancer cell invasion and metastasis; patients with pulmonary adenocarcinoma have a poor prognosis as ACTA2 is highly expressed and distant metastasis is substantially enhanced in cancer cells.^[57]^ Studies have shown that ACTA2 expression can be regulated by the JAK2/STAT1 signaling pathway; abnormal expression of ACTA2 can promote breast cancer invasion and metastasis; in HER2-positive breast cancer, ACTA2 expression is elevated, while no relapse occurs but the survival rate falls sharply.^[58]^ With ACTA2 (α-SMA) being a marker of myofibroblasts in invasive breast cancer, patients with highly expressed ACTA2 are reported to have a markedly reduced overall survival rate.^[59]^ Cancer-associated fibroblasts (CAFs) serve as key players in promoting cancer growth, invasion, and metastasis; the elevated expression of the CAF marker ACTA2 (α-SMA) highly increases the risk of lung cancer metastasis.^[60]^ Likewise, patients with pulmonary adenocarcinoma tend to have a poor prognosis because of the highly expressed ACTA2 in cancer cells and the enhancement of distant metastasis.^[57]^ In different subtypes of ovarian cancer, a high expression level of ACTA2 (α-SMA) is indicative of greater invasiveness of the corresponding subtypes.^[61]^ Most studies have shown that cancer invasion and metastasis are affected by ACTA2, of which a high expression level is associated with a lower survival rate; intriguingly, our study suggested that ACTA2 served as a protective factor in OS, and patients with a high expression level of ACTA2 were shown to have a better prognosis; this underlies the need to further study the mechanism of action of ACTA2 in OS. The lysyl oxidase (LOX) family currently has 5 highly homologous LOX proteins: LOX, LOX-like 1 (LOXL1), LOX-like 2 (LOXL2), LOX-like 3 (LOXL3), and LOX-like 4 (LOXL4); LOXL1 is regarded to have associations with the progression of different types of cancer.^[62,63]^ In colorectal cancer, LOXL1 appears to inhibit cancer growth, invasion, and metastasis through negative regulation of YAP activity.^[64]^ In addition, LOXL1 is reported to inhibit bladder cancer growth by acting on the Ras/ERK signaling pathway.^[65]^ Despite the study results consistent with our analysis of OS, opinions are mixed in cancer research. LOXL1 is regulated by Integrin α11 and involved in the development and progression of non-small cell lung cancer (NSCLC).^[66]^ When upregulated, LOXL1 expression is shown to build up chemotherapy resistance in patients with pancreatic ductal adenocarcinoma^[67]^; chemotherapy resistance is likewise enhanced in NSCLC.^[68]^ All this illustrates the necessity to clarify the specific mechanism of action responsible for the protective effect of LOXL1 in OS in future studies.

Multiple datasets were consolidated and data from different databases were collected for analysis to overcome the limitations of independent studies and improve the reliability and quality of bioinformatics analysis. Despite all this, the present study has its own limitations. First, this study has a small sample size because of the incidence of OS, representing a constraint on the generality of the study results. Second, the 4 protective genes identified in the survival model have not yet been clinically validated, and the specific mechanisms of these genes should be further verified via in vitro and in vivo trials.

## 5. Conclusions

In conclusion, this study identified DEGs related to OS metastasis and survival through bioinformatics analysis and established an ideal prognostic model for OS patients after screening for hub genes to broaden the understanding of OS metastasis and prognosis. In the prognostic model, 4 genes were found to protect OS patients, including TPM1, TPM2, TPM3, and TPM4.

## Author contributions

**Conceptualization:** Zheng Fu, Hongjian Yu

**Data curation:** Zheng Fu

**Methodology:** Zheng Fu

**Visualization:** Zheng Fu

**Writing – original draft:** Zheng Fu

**Writing – review & editing:** Zheng Fu, Guofeng Sun, Jingtian Li, Hongjian Yu
